# Editorial: Innate immunity and renal transplantation

**DOI:** 10.3389/fimmu.2023.1206683

**Published:** 2023-05-17

**Authors:** Qingdi Cheng, Jinhua Zhang, Tong Zheng, Ning Na

**Affiliations:** Department of Kidney Transplantation, The Third Affiliated Hospital of Sun Yat-sen University, Guangzhou, China

**Keywords:** kidney transplantation, innate immunity, ischemia-reperfusion injury, myeloid-derived suppressor cells, neutrophil extracellular traps, complement

After approximately 70 years of development, kidney transplantation has become the most mature and successful of all organ transplant procedures and the optimal therapy for most patients with end-stage renal disease (1, 2). However, despite advances in transplantation techniques, ischemia-reperfusion injury and post-transplant rejection are still major factors affecting long-term transplant survival. Recently, studies have highlighted the role of innate immunity in the pathogenesis of kidney transplant rejection and ischemia-reperfusion injury by initiating a cascade of immune responses (3, 4). Understanding the interaction between innate immunity and kidney transplantation can help identify potential therapeutic targets and improve overall transplant success rates.

This Research Topic was initiated by Ning Na et al. and published in 2022 in Frontiers in Immunology with the aim of providing a new sight of valuable knowledge in innate immune system related to kidney transplantation, and novel new ideas and strategies for the diagnosis and treatment of kidney transplant-related problems. In this special issue, we present 4 original research articles that address such issues, investigating effects of neutrophils in kidney ischemia reperfusion injury, identifying immune characteristics and complement system during rejection, and role of Myeloid-derived suppressor cells (MDSCs) in immune tolerance. Below is a summary of these articles.

Ischemia reperfusion injury (IRI) is an inevitable process after renal transplantation. It is closely related to severe complications such as delayed graft function (DGF), acute rejection (AR) and graft failure. Clinical studies have found that ischemia/reperfusion injury is one of major contributors to delayed graft function and recovery through complement system activation (5, 6). In recent years, bioinformatics analysis and machine learning algorithms have been widely used in clinical and basic research. It has been found that Neutrophil extracellular traps (NETs) play an important role in the process of IRI (7). NETs are extracellular DNA structures modified by a variety of protein substances released by neutrophils in response to strong signaling stimuli. Wu et al. comprehensively analyzed the expression of NET-related genes (NRGs) in IRI, identified gene clusters with different degrees of IRI, and constructed a robust prediction strategy for DGF and long-term graft survival. Microarray and RNA-seq datasets were obtained from the GEO database. Differentially expressed NRGs (DE-NRGs) were identified by differential expression analysis, and two distinct IRI clusters were identified by cluster analysis of IRI samples using NMF algorithm. Machine learning algorithms were used to screen DGF-related hub NRGs, and based on these hub NRGs, robust prediction strategies were constructed for DGF and long-term graft survival. The study provided references for new diagnostic and therapeutic strategies for managing renal IRI, which may reduce the risk of acute rejection post-transplantation.

Rejection is the primary concern after kidney transplantation. Current diagnosis of AR still relies on graft biopsy, and graft injury is not immediately detectable in the presence of AR. Yao et al. investigated the development of sensitive biomarkers for AR diagnosis to protect renal function. Key differentially expressed genes (DEGs) were identified by clinical data and bioinformatics analysis, and the immune characteristics of differentially expressed genes were determined. Aspartate aminotransferase 2 (GOT2) and syntaxin binding protein 3 (STXBP3), which is found to be enriched in circulating innate immune cells like monocytes and dendritic cells, were screened as key DEGs. STXBP3 and GOT2 were confirmed to be highly expressed in AR patients by RT-qPCR, ELISA and IHC staining, which can reflect the immune status of AR patients and have good diagnostic value for early AR.

Complement is an important component of the innate immune system and plays a critical role in the immune response to solid organ injury and rejection. Complement system activation leads to the generation of chemotactic factors, phagocytosis, and lysis of target cells (8). Targeting complement activation have been demonstrated new therapeutic strategies to prevent and treat IRI and rejection. Clinical studies have found that ischemia/reperfusion injury and preformed anti-HLA antibodies are major contributors to delayed graft function and recovery through complement system activation. In previous clinical trials, M101 prevented delayed graft function (DFG), a hallmark of ischemia/reperfusion injury, when administered to renal transplant preservation solutions. Bénédicte Puissant-Lubrano et al. found that M101 can preserve allograft organs from complement-mediated damage in two ways: by maintaining aerobic metabolism, thereby avoiding complement activation, and by preserving organs from injury. These results explain the benefit of M101 in preserving graft quality and avoiding inflammation and graft rejection to protect the graft from ischemia/reperfusion injury and preformed anti-HLA antibodies.

Immune tolerance is a critical mechanism for maintaining long-term allograft survival in kidney transplant recipients. This can be achieved through various approaches, including immunosuppressive medications and stem cell therapy (9). It has been reported that granulocyte and macrophage colony-stimulating factor (GM-CSF) can induce myeloid-derived suppressor cells (MDSCs) to cause immunosuppression *in vitro*. Cao et al. tried to find an effective way to generate more efficient MDSCs for cell therapy. Studies have found that TGF-β combined with GM-CSF can induce a large number of MDSCs with strong immunosuppressive function *in vitro*. MDSCs induced by TGF-β + GM-CSF exerted its immunosuppressive effect mainly by regulating Arg-1 pathway. In addition, the cells are capable to promote significant expansion of Treg cells, thereby participating in the establishment of robust immune tolerance. Therefore, effective treatment of transplant immune rejection by TGF-β + GM-CSF-induced MDSCs cell therapy could form the basis of new clinical strategies.

In summary, in this special edition, there are various findings at different stages of treatment. First, valuable treatment time can be provided clinically through the development of early rejection marker detection and timely prevention. At the same time, it is not difficult to find applications of bioinformatics and emerging machine learning techniques in the study of immunological effects and kidney transplantation, which play a key role in suggesting mechanisms of immunological effects and kidney transplantation. In recent years, the role and mechanisms of various immune cells in kidney transplantation have been gradually revealed and have gradually attracted attention based on the immune rejection of kidney transplantation. Based on the relationship between immune reaction and kidney transplantation, this special issue elaborates on the role and mechanism of innate immunity in kidney transplantation, in order to clarify the mechanism of transplantation immunity in kidney transplantation and explore a new direction for the research of kidney transplantation immunity in the future.

## Author contributions

All authors listed have made a substantial, direct, and intellectual contribution to the work and approved it for publication.
